# Moral awareness and its relationship with moral sensitivity among Iranian nursing students: A basis for nursing ethics education

**DOI:** 10.1002/nop2.1344

**Published:** 2022-09-20

**Authors:** Parvin Rahmani, Mozhgan Behshid, Mehran Seif‐Farshad, Saeid Mousavi, Fatemeh Molaei Tavani

**Affiliations:** ^1^ Student Research Committee Department of Medical‐Surgical Nursing Faculty of Nursing and Midwifery Tabriz University of Medical Science Tabriz Iran; ^2^ Medical Education Research Center, Health Management and Safety Promotion Research Institute, Department of Medical‐Surgical Nursing, Faculty of Nursing and Midwifery Tabriz University of Medical Sciences Tabriz Iran; ^3^ Department of Statistics and Epidemiology, Faculty of Health Tabriz University of Medical Sciences Tabriz Iran

**Keywords:** moral awareness, moral sensitivity, nursing students

## Abstract

**Aim:**

This descriptive‐analytical correlational study was carried out to examine moral awareness and its relationship with moral sensitivity among Iranian nursing students.

**Design:**

A descriptive‐analytical correlational study.

**Methods:**

The present descriptive‐analytical study was performed among 140 Iranian nursing students. The data collection tool was a three‐part questionnaire including (1) Demographic Information Questionnaire, (2) Nurses' Awareness about the Iranian Nursing Code of Ethics by Mohajjel Aghdam and (3) Moral Sensitivity Questionnaire (MSQ) by Kim Lutzen. The data collected from these questionnaires were analysed in SPSS‐26 using descriptive and inferential statistics.

**Results:**

The mean moral awareness (29.42 ± 4.01) was good, and the mean moral sensitivity (135.05 ± 18.79) was moderate among the students. A significant positive correlation was observed between the total score of moral awareness and moral sensitivity (*r* = .22, *p* = .009) in the nursing students.

## 1 INTRODUCTION

The healthcare environment is always fraught with moral issues and challenges for all members of the healthcare team, including nurses as the largest group of service providers in the healthcare system (Rasoal et al., 2017). Due to their continuous attendance to the patients at their bedside, nurses have a major effect on the quality of and patients' satisfaction with healthcare services (Rohde & Domm, 2018; Zamanzadeh et al., 2017). Adherence to ethical principles contributes to the provision of high‐quality nursing care (Rahnavard et al., 2021). Commitment to ethics in healthcare constitutes the core of nursing values (Eskandari et al., 2016), and the World Health Organization (WHO, n.d.) has emphasized the significance of professional behaviour in accordance with healthcare values and ethics (Organization).

Although ethics has long been closely associated with the nursing profession, it has classically received attention since the foundation of the International Council of Nurses (ICN) in 1899, such that the first ethical codes for nurses were compiled and published by ICN in 1953 (Stievano & Tschudin, 2019). The American Nurses Association (ANA) considers ethical codes as part of the principles of nursing profession and mandates ethical commitment in any relationship, even with nursing students (Fowler, 2017; McCrink, 2010).

In clinical practice, nurses experience numerous ethical challenges and conflicts on a nearly daily basis (Hoskins et al., 2018) and must make proper moral decisions about them to meet the patients' needs (Milliken, 2018). Moral awareness is defined as the first important step in the process of making moral decisions and recognizing the ethical consequences of all nursing practices (Milliken & Grace, 2017). That is, nurses must first recognize the potential ethical consequences of their actions to effectively address patients' problems and needs (Milliken, 2018).

By learning about ethics, nurses can promote their professional knowledge and create a suitable physical, social and mental environment for patients (Sadeghi & Alavi, 2018). Zakaria et al. (2016) showed that enhancing nurses' moral awareness with an educational programme promotes their ethical behaviour (Zakaria et al., 2016). In contrast, poor moral awareness disrupts correct decision‐making and leads to a negligence of the ethical aspects of health care (Hoskins et al., 2018). Most nurses believe that awareness about the principles of professional ethics is critical in their profession as healthcare provider (Osingada et al., 2015). Nevertheless, studies report a moderate level of professional moral awareness among nurses and nursing students (Adhikari et al., 2016; Osingada et al., 2015; Sadeghi & Alavi, 2018). Mere awareness of ethics does not guarantee adherence to it in practice; rather, there is a need for adequate moral sensitivity as well (Zakaria et al., 2016). moral sensitivity as a key component of moral decision‐making (Kim & Park, 2019). Ersoy and Göz have described moral sensitivity as the ability to discern the moral dimension of a situation (2001). Moral sensitivity in nursing is defined as the ability to identify moral challenges, having an accurate intellectual and emotional interpretation of vulnerable situations and making decisions about other people with an eye towards the ethical consequences (Ahn & Yeom, 2014). Moral sensitivity refers to self‐awareness about one's responsibilities in critical ethical situations (Lützén et al., 2006). This moral characteristic enables nurses to effectively care for their patients besides helping them understand moral problems in the face of moral issues in their professional environment and make moral decisions for their patients (Yeom et al., 2017). Meanwhile, nurses diminished moral sensitivity leads to their negligence of ethical issues and patients' rights (Nora et al., 2017).

According to the findings of a study by Sadeqi et al. (2018), nurses working at emergency departments had a moderate level of moral awareness, sensitivity and performance, and there was a significant positive correlation among these variables. In their study, moral sensitivity also turned out to be a more important predictor of moral performance than moral awareness. They concluded that moral awareness and sensitivity are inseparable parts of the decision‐making process and can shape nurses' performance (Sadeghi & Alavi, 2018). According to Noh et al. (2013), adherence to moral and legal principles in healthcare settings requires awareness of and sensitivity to these principles and can guarantee the accurate provision of care (Noh et al., 2013). Still, various studies have reported moderate levels of moral sensitivity among nurses (Akca et al., 2017; Dalvand et al., 2017a; Nora et al., 2017; Roshanzadeh et al., 2017) Evidence also suggests that nurses' moral sensitivity greatly affects the quality of care received and the patients' satisfaction (Amiri et al., 2019).

The undergraduate nursing programme in Iran lasts four years, which translates to a bachelor's degree. Undergraduate nursing students, according to the curriculum, learn the basics of nursing professionalism in the form of a course on the “Principles and Skills of Nursing.” In the second semester, they gradually enter the clinical environment in the hospital while passing the course of “Nursing Ethics and Professional Relationships”; and during the following semesters, they learn the clinical courses of nursing along with the ethical issues in each ward. Afterwards, they have to pass a Nursing Ethics and Professional Relationships course and attend seminars and workshops on ethics and thus gain experience in establishing an ethical relationship with the patients/clients, their families and the healthcare team, as well. Until the eighth semester, nursing students gain extensive experience about adherence to ethical principles in their relationship with patients and the healthcare team in the form of clinical training.

The content of the course of “Nursing Ethics and Professional Relationships” includes definition of nursing ethics and its importance, philosophy of ethics, concept of health and spirituality, concept of human dignity and ethical values, history of nursing ethics, four pillars of bioethics, concept of moral sensitivity; communication models of nurses, physicians and patient; principles of moral decision‐making, ethical concepts in nursing regulations, human rights and respect for the client/patient, professional responsibilities in nursing, ethical issues in nursing care for vulnerable groups, ethical codes and professional guidelines, codes of professionalism, ethical and legal problems in nursing (professional misconduct, malpractice, etc.), application of ethical and professional principles in specialty care, challenges of nursing ethics in the clinic and providing solutions based on ethical standards and codes.

To overcome moral challenges in their future role as nurses, nursing students must possess a high level of moral awareness and sensitivity, because they will face numerous ethical challenges in their future workplace and need to provide comprehensive healthcare to patients by detecting and solving these challenges (Yeom et al., 2017). The authors' review of literature did not show any studies conducted to identify the learning needs of students with regard to moral awareness and sensitivity. Furthermore, it is first necessary to assess students' level of moral awareness and sensitivity and, if necessary, to prepare appropriate educational programmes to promote it.

Therefore, the present research was designed to determine moral awareness and its relationship with moral sensitivity among Iranian nursing students. The authors hope that the findings can prove useful in determining nursing students' baseline moral awareness and sensitivity and identifying their educational needs, so that nursing curricula may become more focused on the students' ethical learning needs.

## 2 METHODS

This descriptive‐analytical correlational study was carried out to examine moral awareness and its relationship with moral sensitivity among Iranian nursing students in Nursing School of Tabriz‐northwestern Iran in 2021. The study population consisted of all nursing students studying in the first year, second semester of undergraduate nursing. The inclusion criteria included nursing undergraduate students studying in the School of Nursing and Midwifery of Tabriz‐Iran University of Medical Sciences having successfully completed the first semester and entering the second semester, having not passed any nursing ethics course at the time of the study and being willing to participate in the study. The participants who had filled out the questionnaire more than once or returned incomplete questionnaires were excluded.

There were 143 eligible nursing students all of whom participated in the study. Given that the data could not be collected in person due to the circumstances imposed by COVID‐19, data were collected virtually. First, the examiner briefed the eligible students on the study objectives and its importance as a basis for educational planning of the nursing ethics course and the study method through WhatsApp™. The participants were also assured of the confidentiality of their data and their freedom for withdrawal if they did not want to continue to participate in the study. Furthermore, informed consent was obtained, and the participants were informed of the voluntary nature of participation, were briefed on how to complete and submit the virtual questionnaires and were asked to answer the questionnaires honestly. Then, the researcher sent them the link to the electronic version of the questionnaires on Porsline® through groups created in WhatsApp™ for the second semester nursing students.

This study was approved by the Research Ethics Committee of Tabriz University of Medical Sciences and was registered under the code IR.TBZMED.REC.1399.600. Three questionnaires were administered to accomplish the study objectives as below:

### 2.1 Demographic information questionnaire

This questionnaire examined participants' personal and demographic information, including age, sex, marital status, level of familiarity with the principles and code of ethics in nursing and resources used to gain information about professional ethics.

### 2.2 Questionnaire on nurses' awareness about the Iranian nursing code of ethics

The questionnaire designed and psychometrically assessed by Mohajjel‐Aghdam et al. (2013) on nurses' awareness about the Iranian nursing code of ethics was administered to assess the nursing students' ethical awareness (Mohajjel‐Aghdam et al., 2013). This questionnaire consists of 35 items that examine the five dimensions including *nurses and society*, *nurses and professional commitment*, *nurses and service delivery*, *nurses and healthcare team members* and *nurses and education and research*. Each item is responded with *yes*, *no*, or *I do not know*. The correct response (*yes*) receives 2 points, *I do not know* receives 1 point, and the wrong response (*no*) 0 point. In the re‐coding step, the *yes* response received 1 point and the *I do not know* and *no* responses received 0. The final score ranged from 0 to 35, where 25–36 indicate a good level, 13–24 a moderate level, and 0–12 a weak level of ethical awareness based on the questionnaire guideline. The face and content validity of this questionnaire were confirmed by ten academic staff at Tabriz University of Medical Sciences and the reliability coefficient was obtained as Cronbach's alpha of 0.81. Mohajjel‐Aghdam et al. (2013) reported Cronbach's alpha of 0.85 for their questionnaire, confirming its reliability (Mohajjel‐Aghdam et al., 2013).

### 2.3 Moral sensitivity questionnaire (MSQ)

The 28‐item MSQ developed and psychometrically assessed by Kim Lützén et al. (1994) in Sweden was administered to assess the nursing students' moral sensitivity (Lützén et al., 1994). This tool's items are scored on a seven‐point Likert scale from *totally agree* (7 point) to *totally disagree* (1 point). The total score ranges from 28 to 196, with higher scores indicating higher moral sensitivity. The six dimensions of this questionnaire include *relational orientation, structuring moral meaning*, expressing *benevolence*, *modifying autonomy*, *experiencing moral conflict and* following the *rules*. In this study, 28–84 were taken to indicate low, 85–140 moderate and 141–196 high moral sensitivity.

We obtained its reliability coefficient as Cronbach's alpha of 0.83. Most studies have confirmed the face and content validity and reliability of the MSQ. In Iran Abbaszadeh et al. (2010) reported this questionnaire's validity as 97% (Abbaszadeh et al., 2010) and Imani et al. (2018) in Iran and Nora CRD et al. (2017) in Brazil reported its Cronbach's alpha as 0.80 and 0.84, respectively (Imani et al., 2018; Nora et al., 2017).

The data collected from these questionnaires were analysed in SPSS‐26 using descriptive statistics (absolute frequency distribution and percentage along with central tendency and dispersion indices including mean and *SD*). Inferential statistics (Pearson's correlation coefficient) were also used to assess the correlation between nursing students' moral awareness and moral sensitivity.

## 3 RESULTS

A total of 143 nursing students studying in the second semester of the nursing bachelor's programme at Tabriz‐Iran University of Medical Sciences participated in this study, but three of them were excluded due to returning incomplete questionnaires. Fifty per cent of the participants were female, and 97.9% were single. The participants' age ranged from 18 to 31 years, with a mean of 20.32 ± 1.77 years. Moreover, 79 students (56.4%) were familiar with the principles and code of ethics in nursing. Furthermore, “training in educational centers” (25.3%) and “attending conferences” (3.3%) scored the highest and lowest among the resources used to learn about nursing ethics, respectively.

As shown in Table 1, on average, the students' moral awareness was good (29.42 ± 4.01) and their moral sensitivity was moderate (135.05 ± 18.79). The moral awareness score was good in 126 (90%) and moderate in 14 (10%) of the students, while the moral sensitivity score was moderate in 97 (69.3%) and high in 43 (30.7%) of the students. A significant positive correlation was found between the total score of moral awareness and moral sensitivity (*r* = .22, *p* = .009).

**TABLE 1:** Levels of awareness and moral sensitivity in nursing students of Tabriz‐Iran University of Medical Sciences (*n* = 140)

Moral sensitivity				Moral awareness			
Level of moral sensitivity	Low	Moderate	High	Level of moral awareness:	Good	Moderate	Weak
Frequency	0	97	43	Frequency	126	14	0
Per cent	0	69.3	30.7	Per cent	90	10	0
Mean ± *SD*	135.05 ± 18.79			Mean ± *SD*	29.42 ± 4.01		

Table 2 presents the correlation between sociodemographic characteristics and the scores of moral awareness and sensitivity of nursing students. No significant correlation was found between age and the variables of moral awareness and moral sensitivity (*p* > .05). The mean scores of moral awareness and moral sensitivity were higher in the female compared with the male students, although the difference between them was not significant (*p* > .05). Furthermore, married students had a higher moral awareness than single students (31.33 ± 0.57), while single students had a higher mean moral sensitivity score than their married counterparts (135.24 ± 18.94), but these differences were not significant either (*p* > .05). The students who were familiar with the principles and code of ethics in nursing had higher mean scores of moral awareness and moral sensitivity. Independent *t*‐test showed a significant correlation only between the two variables of familiarity with the principles of nursing ethics and moral awareness (*p* = .00; Table 2).

**TABLE 2:** Demographic characteristics of nursing students and their relationship with knowledge and moral sensitivity (*n* = 140)

Demographic information	*N* (%)	Moral awareness		Moral sensitivity	
		Mean ± *SD*	*p*‐value	Mean ± *SD*	*p*‐value
Gender					
Female	50%	29.62 ± 3.36	.09, *t* = 0.58	135.74 ± 16.64	.16, *t* = 0.43
Male	50%	29.22 ± 4.59		134.37 ± 20.81	
Marital status					
Single	97.9%	29.38 ± 4.04	.14, *t* = −0.83	135.24 ± 18.94	.2, *t* = 0.81
Married	2.1%	31.33 ± 0.57		126.33 ± 5.68	
Familiarity with ethics					
Yes	56.4%	30.68 ± 2.83	.00, *t* = 4.49	136.05 ± 18.32	.85, *t* = 0.71
No	43.6%	27.80 ± 4.70		133.77 ± 19.46	

Table 3 presents the mean scores of the dimensions of moral sensitivity and their correlation with moral awareness in the nursing students. Among the dimensions of moral sensitivity, expressing *benevolence* had the highest mean score (33.95 ± 5.75), whereas *experiencing moral conflict* had the lowest mean score (11.28 ± 3.81).

**TABLE 3:** Moral sensitivity scores and their relationship with moral knowledge in nursing students of Tabriz‐Iran University of Medical Sciences (*n* = 140)

Moral sensitivity scores	Mean ± *SD*	Moral knowledge	
		*r*	*p*‐value
Moral sensitivity	135.06 ± 18.79	.22	.009
*Subscale scores*			
Autonomy	15.58 ± 2.64	.13	.11
Rules	16.0 ± 2.38	.18	.03
Meaning	25.20 ± 5.73	.08	.29
Benevolence	33.95 ± 5.75	.29	.00
Conflict	11.28 ± 3.81	−.15	.07
Relation	33.02 ± 4.81	.28	.001

Pearson's correlation coefficient was used to examine the relationship of the moral sensitivity dimensions with moral awareness. However, Spearman's correlation test was used to examine the dimensions of *following the rules*, *structuring moral meaning* and *expressing benevolence* due to the presence of outliers. According to the results, among the dimensions of moral sensitivity, *relational orientation*, *following the rules* and *expressing benevolence* were significantly and positively but weakly correlated with moral awareness in the nursing students examined (*p* < .05).

## 4 DISCUSSION

This study examined the level of moral awareness and its relationship with moral sensitivity among Iranian nursing students, the findings revealed a good level of moral awareness in the nursing students. The high level of moral awareness among students might result from their general ethics education at high school. Besides, other ways of obtaining information, such as participating in professional ethics workshops, conferences, congresses and even personal studies on the principles of nursing ethics increase moral awareness. In a study by Mohamadi et al. (2017) in Iran, nursing and midwifery staff and students had a moderate level of awareness of the principles of professional ethics (Mohamadi et al., 2017), which is inconsistent with the present study. The difference in results can be explained by different research populations and different conditions of educational settings. Since the interpretation of results depends on the tools used, different tools in studies can yield different results.

This study showed that nursing students have moderate moral sensitivity. Other studies conducted on moral sensitivity in Iranian nursing students have reported similar findings; for instance, Kohansal (2018) and Shamsizadeh (2020) in Iran also reported a moderate level of moral sensitivity among nursing students (Kohansal et al., 2018; Shamsizadeh et al., 2020). Similarly, studies in other countries, including those by Akca (2017) in Turkey, Alnajjar and Abou Hashish (2021) in Saudi Arabia and Lutzen (2017) in Sweden have also reported a moderate level of moral sensitivity in nursing students, which is in line with the present findings (Akca et al., 2017; Alnajjar & Abou Hashish, 2021; Tuvesson & Lützén, 2017). This consistency can be explained by the similarity of the research population and the similar tools used for measuring moral sensitivity. The moderate moral sensitivity of first‐semester nursing students indicates that they are familiar with the ethical principles of healthcare provision to some extent but are not yet morally sensitive enough due to certain barriers.

Tazakori et al. (2018) reported nurses' moral sensitivity as high, which is not consistent with the present study (Tazakori et al., 2018). Nurses are sometimes not sensitive enough to moral issues due to a variety of reasons and factors (Lee et al., 2017). Lutzen considered factors such as culture, religion, education, age, gender, work experience, upbringing, working conditions and type of education affecting moral sensitivity (Lützén et al., 2006).

In this study, the nursing students' moral awareness and moral sensitivity were significantly and positively correlated, since the students' moral sensitivity increased with their moral awareness. Our review of literature did not show any studies that had assessed the relationship between these two variables among nursing students; however, Sadeghi and Alavi (2018) had examined the relationship among moral awareness, sensitivity, and performance in nurses working in the emergency departments of Alborz Province (Iran) and reported a significant correlation between moral awareness and sensitivity, between moral awareness and performance and also between moral sensitivity and performance (Sadeghi & Alavi, 2018).

Nursing students, who are the nursing workforce of the future, will be exposed to the challenging ethical conditions of nursing in their workplace; they must be morally aware enough to provide wholistic care based on correct moral decision‐making skills (Muramatsu et al., 2019). As such, a merely good awareness of moral issues is not enough, and nurses must also possess correct resources from which to derive their values and based on which to perform moral actions, which can be accomplished by getting more sensitive to moral principles (Amiri et al., 2018; Yeom et al., 2017).

In the present study, among the dimensions of moral sensitivity, the highest score pertained to *expressing benevolence*, which is in line with the results reported by Asmaningrum in Indonesia, Farahaninia and Aghajani in Iran in nursing students (Asmaningrum & Tsai, 2018; Ekramifar et al., 2018; Jalili et al., 2020). Nursing students' higher sensitivity to the domain of benevolence may be because, contrary to nurses, they are not yet in charge of independent healthcare provision and therefore pay more attention to theoretical concepts and their application in clinical settings. The *expressing benevolence* dimension of the MSQ denotes honesty, patient–nurse trust, attention to the patients' reactions to health care, and the patients' insight into and awareness about their disease. Consequently, establishing appropriate and compassionate relationship, along with honesty and benevolence, provides the basis for the trust and confidence of patients and nurses.

The students received a low score in the dimension of *experiencing moral conflict*, which is consistent with the results reported by Abou Hashish in Saudi Arabia (Alnajjar & Abou Hashish, 2021) and Amiri and Sharifi in Iran (Amiri et al., 2018; Sharifi et al., 2020). The low mean score of this dimension indicates that if nursing students do not acquire the skills and abilities needed for identifying moral conflicts and determining the proper way to solve them over the course of their education in university, they may face troubles in solving moral conflicts and overcoming moral challenges in clinical practice and may not show the right reaction to them or may even end up lacking enough power to properly deal with them. Finding a correct moral solution is one of the challenging issues for many nurses in the workplace. Before graduation, nursing students must be able to manage the moral issues related to their profession and distinguish personal from professional values. After graduation, they will be in charge of providing care to patients and will play a major role in their recovery and improved health. Therefore, they must be trained for accepting healthcare responsibilities and understanding, identifying and properly dealing with moral dilemmas.

Among the studied demographic variables, only familiarity with the principles of nursing ethics was significantly and positively correlated with the moral awareness score, as this score was higher in the students who were familiar with these principles. This finding was not unexpected because familiarity with the principles of nursing ethics can effectively increase the moral awareness of nurses and make them to behave better in the face of professional moral issues.

Meanwhile, no significant relationship was found between the other demographic variables and moral awareness and moral sensitivity. Similarly, Mohamadi et al. (2017) found no significant correlation between variables of age, sex and marital status and moral awareness in nurses (Mohamadi et al., 2017). Kohansal and Faraj pour et al. also reported similar results between moral sensitivity of nursing students and the variables of age, sex and marital status, which is consistent with this study (Kohansal et al., 2018; Mostafavian et al., 2019).

Pearson's correlation test revealed that the students' age correlated significantly and positively with the dimension of *following the rules*, and older students scored higher in this dimension. Abbaszadeh and Shirzadegan et al. also reported a direct relationship between nurses' age and the dimension of *following the rules* (Abbaszadeh et al., 2010; Dalvand et al., 2017b). It therefore seems that as students become older, they gain more awareness of the key role of ethics in nursing care and their own professional and ethical responsibilities and thus try to adhere to organizational rules.

### 4.1 Limitations

This study was the first study in Iran that measured nursing students' levels of moral awareness and moral sensitivity, and their relationship. However, one of the limitations of the present study was that the samples were taken from one nursing school, so the results cannot be generalized to nursing students in other areas. Also, samples were selected by non‐random sampling method (census), while in case of random sampling, the sample could be a better example of the target population. Therefore, the researchers suggest conducting studies in different statistical populations and other disciplines of medical sciences by random sampling for more reliable generalizability.

## 5 CONCLUSION

The present findings demonstrated a good level of moral awareness and moderate moral sensitivity among Iranian nursing students in Nursing School of Tabriz‐northwestern Iran in 2021. This finding suggests that similar to nursing students in other universities, these students need more ethics training. The students' moral awareness and moral sensitivity were also correlated significantly and positively. That is, moral sensitivity increased with moral awareness. Educational policymakers and managers should offer educational programmes starting from the beginning of the nursing programme to promote professional ethics among nurses and should also emphasize the development of the various dimensions of moral sensitivity in their curricula. Since the students received the lowest score in the dimension of *experiencing moral conflict*, it is necessary to ensure that nursing students can identify moral conflicts and find appropriate ways to solve them. Effective educational planning is needed to enhance nursing students' moral sensitivity before they take up new responsibilities in clinical settings and officially begin their work as nurses, so as to ensure that they will become successful nurses.

## AUTHOR CONTRIBUTIONS

Mozhgan Behshid and Mehran Seif‐Farshad did the study conception. Mozhgan Behshid and Parvin Rahmani designed the study. Parvin Rahmani and Fatemeh Molaei Tavani collected and involved in provision of the data. Saeid Musavi and Parvin Rahmani analyzed the data. Mozhgan Behshid, Mehran Seif Farshad, Parvin Rahmani, and Fatemeh Molaei Tavani contributed to the interpretation of the data. Mozhgan Behshid and Parvin Rahmani prepared the first draft, critically revised the manuscript, and prepared the final edit. All authors approved the final manuscript and agreed to be accountable for all aspects of the work.

## FUNDING INFORMATION

This study was approved and supported by the Deputy for Research and Technology of Tabriz University of Medical Sciences.

## CONFLICTS OF INTEREST

The authors declare that they have no conflicts of interest with regard to this study.

## ETHICS STATEMENT

This research project was approved by the Ethics Committee of Tabriz University of Medical Sciences (IR.TBZMED.REC.1399.600) and was carried out in compliance with the ethical principles outlined in the Helsinki Declaration. Participants signed an informed consent form before taking part in the study.

## Data Availability

The datasets generated and analyzed during the current study are available from the corresponding author upon reasonable request.
